# Trocar site hernia after gastric sleeve

**DOI:** 10.1007/s00464-021-08787-2

**Published:** 2021-10-26

**Authors:** Sandra Ahlqvist, Axel Edling, Magnus Alm, Johan Blixt Dackhammar, Pär Nordin, Yücel Cengiz

**Affiliations:** 1 Department of Surgical and Perioperative Sciences, Umeå University, Umeå, Sweden; 2 Granlo Health Center, Sundsvall, Sweden; 3 Department of Radiology, Sundsvall Hospital, Sundsvall, Sweden; 4 Department of Anesthesia and Intensive Care, Region Västernorrland, 871 85 Härnösand, Sweden

**Keywords:** Trocar site hernia, Port site hernia, Incisional hernia, Complications, Laparoscopy, Bariatric surgery

## Abstract

**Background:**

Laparoscopy is common in abdominal surgery. Trocar site hernia (TSH) is a most likely underestimated complication. Among risk factors, obesity, the use of larger trocars and the umbilical trocar site has been described. In a previous study, CT scan in the prone position was found to be a reliable method for the detection of TSH following gastric bypass (LRYGB). In the present study, our aim was to examine the incidence of TSH after gastric sleeve, and further to investigate the proportion of symptomatic trocar site hernias.

**Methods:**

Seventy-nine patients subjected to laparoscopic gastric sleeve in 2011–2016 were examined using CT in the prone position upon a ring. Symptoms of TSH were assessed using a digital survey.

**Results:**

The incidence of trocar site hernia was 17 out of 79 (21.5%), all at the umbilical trocar site. The mean follow-up time was 37 months. There was no significant correlation between patient symptoms and a TSH.

**Conclusions:**

The incidence of TSH is high after laparoscopic gastric sleeve, a finding in line with several recent studies as well as with our first trial on trocar site hernia after LRYGB. Up to follow-up, none of the patients had been subjected to hernia repair. Although the consequence of a trocar site hernia can be serious, the proportion of symptomatic TSH needs to be more clarified.

Laparoscopy is common in abdominal surgery, with advantages such as reduced length of hospital stay and less postoperative pain compared to open surgery [1, 2]. A complication to both laparoscopic and open surgery is incisional hernia, and obesity is a risk factor [3]. After a midline incision, up to 10–20% of patients can develop a hernia [4], and approximately 20% of these hernias need surgery [5]. The reported incidence of trocar site hernia has been low at up to 5% [3, 6], but in recent years papers reporting up to 39% have been published [7, 8]. The reported rates vary with study design and follow-up time [6], but—importantly—also the focus on hernia repair in contrast to focus on patients with a history of laparoscopy [9]. Moreover, the method of hernia detection differs. In obese patients, clinical examination of trocar sites for hernias has proven to be unreliable [10–12]. In a study from 2017 we concluded that CT in the prone position is a reliable method for the detection of TSH in the obese [11], and although a small sample size, and all asymptomatic, we found a surprisingly high incidence of TSH after LRYGB. Unfortunately, we have not yet seen publications of this method from other author groups.

TSH can cause significant suffering and a need for surgery [13], but the proportion of symptomatic trocar site hernia is relatively unknown [14].

Obesity is an increasing problem in large parts of the world. Bariatric surgery is gaining in popularity [15]. Gastric sleeve has taken the lead over gastric bypass as the dominating method of choice. Besides obesity, other known risk factors for TSH are the use of larger (> 10 mm) trocars and trocar placement at the umbilicus [3, 8, 16]. In gastric sleeve, the specimen is usually retrieved through the umbilical trocar site which most likely increases the abdominal wall defect. That may increase the risk of TSH in a group of patients already at risk of this complication.

The main objective for this study was to evaluate the incidence of TSH after gastric sleeve, using CT in the prone position. Further, the proportion of patients with symptoms associated to TSH was assessed.

## Materials and methods

79 patients subjected to laparoscopic gastric sleeve in 2011–2016 within the region of Västernorrland, Sweden, were included. The start of inclusion was chosen to when the standard bariatric procedure in our region began shifting from gastric bypass towards gastric sleeve. At follow-up, all patients were examined for TSH using CT scan in the prone position with the abdomen positioned at the center of a ring. A low dose protocol without intravenous contrast was used. The radiological definition of TSH was any intra-abdominal content protruding beyond the peritoneum or the presence of a hernia sac [11, 17]. The CT images were evaluated by an experienced radiologist.

At the index operation, the Hasson technique [18] was used for abdominal entry in the midline, at the umbilicus. This technique is well standardized, and its use is specifically documented in the patient records. Blunt 5 mm trocars were inserted at all four port sites but in the epigastric one, which had a 10–12 mm camera trocar, and the umbilical one, where a 15 mm trocar was used. The specimen was always retrieved through the umbilical trocar site. The aponeurosis of trocar sites > 10 mm was closed by a continuous technique with a slowly absorbable monofilament suture (Polydioxanone, PDS II, Ethicon GmBH, Germany). All patients were given preoperative antibiotic prophylaxis.

Patient data that were obtained from the quality register Scandinavian Obesity Surgery Registry (SOReg), from the patient medical record and from the surgical procedure program Orbit (version 4 and 5): sex, age, pre -and postoperative BMI, pre- and postoperative abdominal circumference, the presence of Diabetes Mellitus, arterial hypertension, chronic obstructive pulmonary disorder, sleep apnea and smoking habits. The comorbidities are not further defined in SOReg. Peri- and postoperative data: duration of surgery, complications (postoperative wound infection, bleeding during surgery, reoperation) noted in the 1-, 2- and when possible 5-year follow-ups, other abdominal surgical procedures pre- and post the gastric sleeve. In the cases where other surgical procedures were found, the surgeon´s documentation of the surgery and outpatient visits were reviewed for description of any hernias, operation technique and the presence of peri- or postoperative complications. For all patients, any documenting of abdominal hernia at the clinical examination pre-surgery was reviewed, as were any postoperative clinical or radiological findings of hernia in a patient not operated since the index operation.

All patients were sent a digital survey for evaluating symptoms related to TSH (Table 1). The patients had not been informed about the presence or not of a hernia at the time of them answering the survey.

**Table 1:** Survey regarding TSH symptoms

Questions			
Do you have any discomfort from the incisions?	Yes	No	
Level of discomfort	0–6^a^		
Level of pain	0–6^b^		
Do you believe that you have a trocar site hernia?	Yes	No	
Have you sought medical advice because of discomfort caused by the gastric sleeve procedure?	Yes		
For what kind of discomfort did you seek medical advice?	Pain	TSH	Other
Are you satisfied with the results of your gastric sleeve?	Yes	No	

^a^0 = No discomfort; 1 = Discomfort easily ignored; 2 = Discomfort not easily ignored but does not affect daily activities; 3 = Discomfort not easily ignored and affects daily activities; 4 = Discomfort that prevents most daily activities; 5 = Discomfort that requires bed rest; 6 = Discomfort that requires immediate help

^b^0 = No pain; 1 = Pain easily ignored; 2 = Pain not easily ignored but does not affect daily activities; 3 = Pain not easily ignored and affects daily activities; 4 = Pain that prevents most daily activities; 5 = Pain that requires bed rest; 6 = Pain that requires immediate help

Statistical analyses were performed using SPSS software (IBM SPSS Statistics version 26). Continuous variables are described as medians and interquartile ranges or means and standard deviations, according to their distribution. Categorical variables are described as absolute numbers and percentages. The two-tailed unpaired t-test or the Mann–Whitney test was used to compare continuous variables and the Chi-square or Fisher’s exact test was used to compare categorical variables, as appropriate. The 5% level was considered as significant.

This study was approved by the Regional Ethical Board of Umeå University (Registration Number 2012/419-31 and 2021-00882) and by the local Radiation Committee. Informed, written consent was obtained from all individual participants included in the study.

## Results

The incidence of trocar site hernia was 17 out of 79 (21.5%), all at the umbilical trocar site. One patient had a hernia in a lateral trocar site as well as in the umbilical one, the lateral TSH not being included in the statistics. The mean follow-up time was 37 months. Patient characteristics are listed in Table 2. There was no significant difference between TSH patients and patients with no TSH regarding baseline characteristics, operative data or comorbidities, apart from a higher prevalence of sleep apnea in the TSH group (p < 0.02). BMI did not differ significantly for patients with and without sleep apnea. The correlation of risk factors to TSH was not analyzed further due to the limited number of patients for each risk factor.

**Table 2:** Patient characteristics (stratified by trocar site hernia on CT)

Variable	TSH (*N* = 17)	No TSH (*N* = 62)	Total (*N* = 79)	*p* value
Sex				0.519^a^
Female	12 (70.6)	49 (79)	61 (77.2)	
Male	5 (29.4)	13 (21)	18 (22.8)	
Age at surgery (years)				
Mean (SD)	44.4 (6.5)	40.6 (9.8)	41.4 (9.3)	0.216^b^
Preoperative BMI (kg/m2)				
Median (IQR)	45.5 (13.3)	43.5 (7)	43.6 (8.3)	0.183^b^
BMI loss (kg/m2)				
Median (IQR)	10.2 (6.8)	10.0 (5.8)	10.1 (6.2)	0.886^b^
* Missing*			*6*	
Patients with previous abdominal surgery	2 (11.8)	19 (30.6)	21 (26.6)	0.213^a^
Patients with later abdominal surgery	0	6 (9.7)	6 (7.6)	0.331^a^
Patients with previously suspected abdominal wall hernia	2 (11.8)	1 (1.6)	3 (3.8)	0.115^a^
Follow-up time (months) Mean (SD)	34.7 (13.2)	37.3 (13.3)	36.7 (13.2)	0.465^c^
Comorbidities				
Hypertension	7 (41.2)	17 (27.4)	24 (30.4)	0.372^a^
Diabetes mellitus	2 (11.8)	5 (8.1)	7 (8.9)	0.639^a^
Sleep apnea	4 (23.5)	2 (3.2)	6 (7.6)	0.018^*,**a**^
Chronic obstructive pulmonary disorder			0	
Smoking status	0	3 (4.1)	3/74 (4.1)	1.000^a^
* Missing*			*5*	
Operative data				
Duration of surgery (hours)				
Mean (SD)	0.40 (0.16)	0.38 (0.12)	0.38 (0.13)	0.584^c^
Perioperative bleeding complications	0	2 (3.2)	2 (2.5)	1.000^a^
Postoperative wound infection	1 (6.3)	0	1/76 (1.3)	0.211^a^
* Missing*			*3*	
Reoperation			0	
TSH at 1-year clinical follow-up			0	
* Missing*			*3*	
TSH at 2-year clinical follow-up			0	
* Missing*			*13*	

Data are presented as numbers (percentages) of patients unless otherwise specified

*IQR* interquartile range, *SD* standard deviation, *TSH* trocar site hernia

^a^Fisher’s exact test

^b^Mann-Whitney test

^c^Two-tailed, unpaired T-test

*Significance level *p* < 0.05

The response rate of the survey regarding TSH symptoms was 71 out of 79 (89.9%) patients. There was no significant correlation between patients’ subjective symptoms and the presence of a TSH at CT examination (Table 3). Of the patients having a TSH, none experienced any discomfort from the trocar incision sites. Three patients believed they had a trocar site hernia, but none of them sought medical advice for that reason.

**Table 3:** Survey answers—symptoms and trocar site hernia

	TSH *N* = 16	No TSH *N* = 55	Total *N* = 71	*p* value
Discomfort from the incisions^a^	0	6 (10.9)	6 (8.5)	0.326^d^
Belief in having a TSH	3 (18.8)	4 (7.3)	7 (9.9)	0.185^d^
Have sought medical advice because of discomfort caused by the GS procedure^b^	2^c^ (12.5)	8 (14.5)	10 (14.1)	1.000^d^
Discomfort from the insicions^a^ + Belief in having a TSH	0	3 (5.5)	3 (4.2)	0.143^d^
Belief in having a TSH + Have sought medical advice because of discomfort caused by the GS procedure^b^	1^c^ (6.3)	2 (3.6)	3 (4.2)	1.000^d^
Discomfort from the incisions^a^ + Have sought medical advice because of discomfort caused by the GS procedure^b^	0	4 (7.3)	4 (5.6)	0.467^d^

Data are presented as numbers (percentages)

*TSH* Trocar site hernia, *GS* gastric sleeve

^a^Any discomfort (0–6)

^b^Any type of discomfort (pain;TSH;other)

^c^These patients sought medical advice because of pain, not specified to pain from TSH

^d^Fisher’s exact test

## Discussion

Trocar site hernia (TSH) refers to an incisional hernia occurring at the trocar incision site after a laparoscopic procedure. Laparoscopy is frequently used in various specialties for abdominal surgery such as general surgery, urology and gynecology, and patients of various ages and comorbidities may be subjected to a laparoscopy. The reported TSH incidence could be underestimated, since most papers refer to symptomatic cases only, or have not had the specific intent to detect a TSH. Only a few studies have used specific imaging for detection of TSH, but they report high incidences [10, 11, 16, 19]. Especially in obese patients, a small TSH embedded in thick subcutaneous fat is difficult to detect clinically [11, 12]. After a postoperative weight loss, the scar after the skin incision may not correlate to the site where the abdominal wall was penetrated. In the present study, we used CT scan in the prone position with the abdomen positioned at the center of a ring. The rationale for this is to imitate a constant Valsalva, and to allow a possible hernia to protrude freely. This method has a high interobserver-agreement [11]. Although the method seems robust and easy to implement in a clinical setting where CT is available, we are surprised to find that no other reports of its use have been published. The use of sonography for detection of abdominal wall defects, as recommended by the European Hernia Society, has been shown to be a valid alternative to CT [20] as it is less costly and lacks patient irradiation. On the other hand, in an obese patient a CT may be less user dependent and more specific.

In our previous study on TSH after a LRYGB (laparoscopic Roux-en-Y gastric bypass), all hernias were located at the umbilical trocar site. In that study several of the lateral ports were larger than 5 mm. The present study has an almost four times larger study population but otherwise similar patient characteristics, methodology and follow-up time. Although probably not highly likely, the ring to lay upon may have covered the most lateral trocar sites, preventing hernias from protrusion, and we have chosen to keep the number of TSHs without false negatives and therefore not included the one lateral TSH in the statistics. The higher incidence of TSH after gastric sleeve may be explained by that the removed stomach is retrieved through the 15 mm umbilical trocar site, possibly further enlarged due to this manipulation. This strongly indicates that a sleeve patient is more likely to develop a TSH than a LRYGB patient. That most trocar site hernias are found at the umbilicus is again striking and needs to be further addressed. The umbilical site has previously been shown to constitute a higher risk for hernia formation, as are larger trocars [6, 8, 13]. One study report that excessive manipulation of the trocar site to remove specimens during surgery was an important risk factor for TSH [21]. A randomized control trial comparing TSH incidence between a LRYGB and a gastric sleeve is necessary. Furthermore, as development of a TSH might be correlated to an insufficient closure technique, this is another matter worth further scientific evaluation. In fact, to date we do not know whether the principals of optimal closure techniques applied for open surgery is advised also for trocar incisions. In conclusion, regarding an initial trocar site in the umbilicus versus outside of the umbilicus, there should be further comparing studies on initial trocar site and the aspects of feasibility and safety in creating the site and extracting a large specimen, but also on the best closure techniques.

Karampinis et al. reports in a meta-analysis that studies with a follow-up after 12 months had significantly higher TSH incidence compared to a follow-up less than 12 months [9]. Our follow-up time of a mean of 37 months is as far as we know among the longest follow-ups reported. Several risk factors for TSH have been described. They can be related to patient factors (older age, smoking status, comorbidities as obesity, diabetes) [3, 8, 9, 22], to technical and perioperative factors (duration of surgery, trocar size) [22, 23] and to postoperative factors such as wound infection [8]. In our material, sleep apnea was the only variable significantly more seen in patients with TSH than patients without. Sleep apnea could be speculated to be more common with higher BMI, and obesity as a TSH risk factor is often described. But only six patients had this condition, there was no significant difference in BMI for patients with and without sleep apnea, nor did BMI differ significantly for TSH and no TSH patients. Some variables were not included in the analyses because of missing data (abdominal circumference, 1- 2 and 5-year routine clinical follow-ups) or lack of reliable journal sources (for example factors associated with impaired wound healing such as anemia, American Society of Anesthesiologist (ASA) score), and for other variables the numbers were too small to draw meaningful conclusions (chronic obstructive pulmonary disorder). This limitation to our study is partly due to the sample size, and partly to the retrospective collection of data in contrast to a study design where all variables are chosen specifically for the purpose of conducting a TSH study.

Another limitation is the potential of bias in symptomatic patients, as all patients were aware of the purpose of the study when accepting inclusion, but this is in part contradicted by the fact that so few of them reported symptoms of TSH or own belief in that they had this condition. Perhaps the main limitation is the large number of our patients having been subjected to abdominal surgery prior to or after the bariatric procedure, thereby making our results not undeniably connected to the gastric sleeve. Of the 21 patients with previous abdominal surgery (Table 2), six had midline incisions and none of them had a hernia in our study, but two hernias (both at the umbilical trocar site) were found in patients with previous laparoscopy. Also, there may have been undetected preexisting umbilical hernias in previously non-operated patients. In three patients, an umbilical hernia was suspected at clinical examination documented in the patient journal at any time previous to the gastric sleeve, none of them had any mentioning of umbilical hernia in the surgeon’s documentation from the gastric sleeve operation. The reason these patients were included was the large difficulty in obtaining enough patients willing to participate to maintain an adequate power. To partly account for these limitations, careful reviewing of relevant medical records, including the surgeon’s documentations of procedures, descriptions of clinical examinations and imaging have been made, for as full disclosure as possible. Procedures not engaging the upper abdomen, umbilical region or midline are not reported in the study.

The clinical relevance of these hernias is due to the fact that they can lead to significant morbidity such as bowel obstruction and strangulation and require acute surgery [13, 24, 25]. But the proportion of the symptomatic cases is poorly understood [22]. None of the 79 patients in our study had up to follow-up been subjected to hernia repair. In the survey sent out to the patients asking them to answer questions regarding their experience of TSH related symptoms, only a few reported discomfort or pain from the trocar sites, and there was no significant correlation of those who did to those with TSH at CT. Nevertheless, our results of an alarming 21.5% incidence of TSH are in line with other recent data, and bearing in mind the high number of laparoscopic procedures performed, even a small proportion of symptomatic TSH can amount to a significant patient suffering and strain on the welfare systems. To further understand the relevance of symptomatic TSH and TSH repair, we are presently conducting a larger register study of more than forty thousand patients laparoscopically operated for obesity in Sweden.

## Conclusions

The incidence of TSH is high after laparoscopic gastric sleeve, a finding in line with several recent studies as well as with our first trial on trocar site hernia after LRYGB. Up to follow-up, none of the patients had been subjected to hernia repair. Although the consequence of trocar site hernia can be serious, the proportion of symptomatic TSH needs to be more clarified.
